# Multiple Ethnic Origins of Mitochondrial DNA Lineages for the Population of Mauritius

**DOI:** 10.1371/journal.pone.0093294

**Published:** 2014-03-27

**Authors:** Rosa Fregel, Krish Seetah, Eva Betancor, Nicolás M. Suárez, Diego Calaon, Saša Čaval, Anwar Janoo, Jose Pestano

**Affiliations:** 1 Department of Genetics, Faculty of Medicine, University of Las Palmas de Gran Canaria, Las Palmas, Spain; 2 Department of Genetics, Faculty of Biology, University of La Laguna, La Laguna, Spain; 3 Department of Anthropology, Stanford University, Stanford, California, United States of America; 4 Forensic Genetics Laboratory, Institute of Legal Medicine of Las Palmas, Las Palmas, Spain; 5 IDEAS Interdepartmental Centre, Ca'Foscari University, Venice, Italy; 6 Department of History, University of Mauritius, Reduit, Mauritius; University of Oxford, United Kingdom

## Abstract

This article reports on the first genetic assessment of the contemporary Mauritian population. Small island nodes such as Mauritius played a critical role in historic globalization processes and revealing high-resolution details of labour sourcing is crucial in order to better understand early-modern diaspora events. Mauritius is a particularly interesting case given detailed historic accounts attesting to European (Dutch, French and British), African and Asian points of origin. Ninety-seven samples were analysed for mitochondrial DNA to begin unravelling the complex dynamics of the island's modern population. In corroboration with general demographic information, the majority of maternal lineages were derived from South Asia (58.76%), with Malagasy (16.60%), East/Southeast Asian (11.34%) and Sub-Saharan African (10.21%) also making significant contributions. This study pinpoints specific regional origins for the South Asian genetic contribution, showing a greater influence on the contemporary population from northern and southeast India. Moreover, the analysis of lineages related to the slave trade demonstrated that Madagascar and East Asia were the main centres of origin, with less influence from West Africa.

## Introduction

Since prehistoric times, the Indian Ocean has provided the backdrop for major maritime expansion events, resulting in the exchange of crops, stocks and languages among African, South Asian and Island Southeast Asian populations, and catalysing extensive cross-cultural interaction [1].

At the western edge of the Indian Ocean, African and Austronesian communities inhabited Madagascar and the Comoro Archipelago before European colonization. Other islands in this region, such as Réunion Island and Mauritius, although visited by the Arabs during the Islamic expansion, were not permanently occupied until the arrival of Europeans. Consensus suggests that in all cases, Europeans first imported sub-Saharan African and Malagasy slaves for labour provision and later, indentured workers from South and Southeast Asia [2]. However, the complexity of this situation is highlighted by historical interrogations of extant records [2]. The present day multi-ethnic population structure of these islands reflects their idiosyncratic histories.

Early molecular genetic studies of the haploid characteristics of mitochondrial DNA (mtDNA) have been used to confirm the presence of Austronesian female lineages in Madagascar [3]. Subsequent research revealed the complex nature of settlement on this island, with the inclusion of at least three putative parental populations of African, Indonesian and Indian origin [4], [5], [6], [7]. A similar result was obtained for the Comoro Archipelago [8], [9]. In relation to Réunion Island, phylogeographic analysis not only detected strong founder effects and gender asymmetrical gene flow [10] but also tracked the most probable origins of the *Malbar* and *Zarab* ethnic groups [11].

This study initiates a process to disentangle the mtDNA genetic composition of Mauritius. Together with Réunion and Rodrigues, these islands form the Mascarene Archipelago, situated approximately 700 km off the eastern coast of Madagascar. From a genetic point of view, Mauritius is particularly interesting as it underwent complex and multi-scalar processes of European colonization. Visited by the Portuguese in 1510, the first permanent settlement was by the Dutch in 1638. The number of Dutch settlers was low; however, they brought hundreds of slaves to the island, mainly of Malagasy origin. A century later the Dutch abandoned the island with a contingent of their slaves. As with other enclaves marked by slavery, runaways were common and the Dutch left a group of such *maroons* on the island upon their departure. During the next century, the French occupied Mauritius. Slaves accompanied the first French settlers and grew steadily in size during the 18th century, reaching 75–85% of the island's population [2]. These slaves were mainly imported from Madagascar and Mozambique and it was estimated that 160,000 slaves were brought to Mauritius and Réunion between 1670–1810: 45% being Malagasy, 40% East African, 13% Indian and 2% West African [12].

In 1810, the British captured Mauritius. Although this coincided with the Act of Abolition of the Trade in Slaves, it is suspected that some 30,000 slaves were imported to the island by the early 1820 [2]. In spite of this illegal trade, the emancipation of slaves and the high mortality rate due to cholera and malaria epidemics reduced the number of men capable of heavy agricultural work. This led to the “Great Experiment”, a trail by the British to use imported indentured labour, mainly from India. The situation is far from simple as African indentured labourers, as well as other Asian ethnic groups, were also recruited throughout this period [2]. By 1846, Indian immigrants represented more than 35% of the population; by 1871 this figure had risen to two thirds, a proportional representation that has remained constant to present day [2]. The partial admixture of these groups gave rise to the contemporary Creole population, and a highly plural society that retains features, such as polyglot language traditions, from its diverse founder populations.

In the present research we use the non-recombining characteristics of the mtDNA molecule and its well-known phylogeographic structure to identify the maternal sources and present day proportions of this admixed population.

## Materials and Methods

### Ethics Statement

Ethical approval was provided by the Truth and Justice Commission, Port Louis, Mauritius, Chaired by Prof. Alex Borain. Written consent was recorded from all participants prior to partaking in the study. Consent was documented on a form, copies of which were lodged with the Truth and Justice Commission, Mauritius. The ethics committee at the Truth and Justice Commission approved, sanctioned and fully endorsed this mode of consent recording.

### Samples

**Figure 1:**
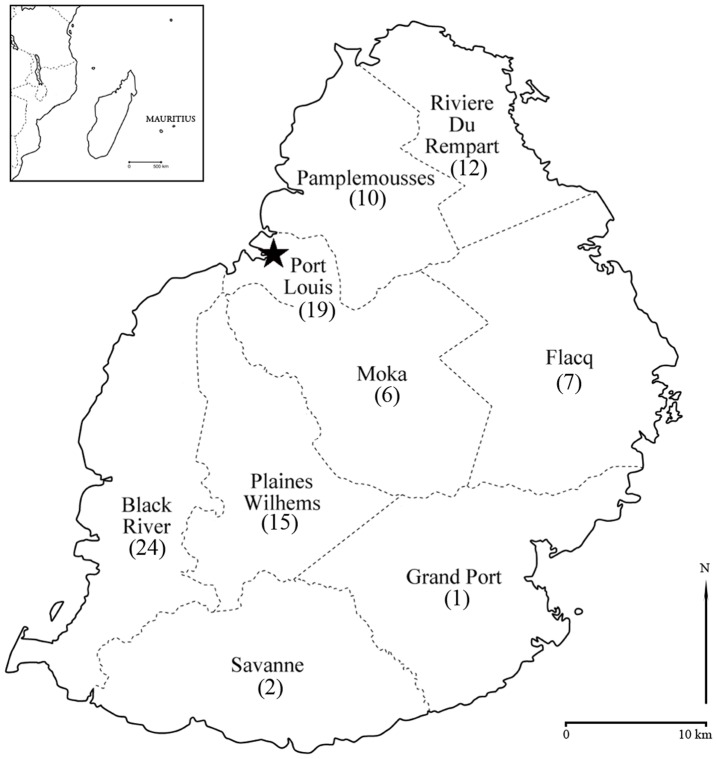
Map showing the geographical origin of samples. Mauritius, with the capital, Port Louis, starred. Figures in brackets represent number of individual samples taken per district.

Ninety-seven samples were taken from anonymous unrelated Mauritian donors and all collaborators gave their informed consent to this project. These miscellaneous samples derived from all geographic zones on the island (Figure 1). Ethnic affiliation and place of birth were obtained in order to determine the suitability of the sample for reflecting the whole Mauritian diversity.

For comparative purposed, mtDNA sequences from Mauritian putative parental populations were retrieved from published data: Madagascar (n = 170), South Asia (n = 6504), East Asia (n = 9074), Southeast Asia (n = 4293), Africa (n = 9780) and Europe (n = 2913). Samples from Austronesia (n = 4451) and Réunion Island (n = 303) were also obtained (Table S1; Table S2). For interregional comparison within South Asia, the Indian samples were subdivided in five distinct geographical areas: North, Southwest, Southeast, West and East. For assessing the geographical origins of lineages related with the transatlantic slave trade, the African samples were classified into West Africa, West Central Africa and East Africa groups.

### MtDNA analysis

Samples consisted in all cases of buccal swabs. DNA extraction was carried out using QIAmp DNA Mini Kit (QIAgen), following manufacturer recommendations. The complete hypervariable region (HVR) was amplified using the primers L15676 and H00945, previously described by Maca-Meyer et al.[13]. The PCR was carried out in 50-μl volumes, containing 1X Tris–HCl buffer, 200 μM of each dNTP, 2.5 mM MgCl2, 50 pmoles of each primer and 3 U of Taq polymerase (Bioline). The amplification was carried out in an Applied Biosystems 2720 Thermal Cycler with the following conditions: 30 amplification cycles with denaturation at 94°C for 30 s, annealing at 55°C for 30 s, and extension at 72°C for 90 s.

HVR haplotypes were classified into haplogroups based on the mtDNA tree (Build 15) [14]. For those haplotypes that could not be classified based solely on HVR sequence, several SNPs (1473, 1888, 3432, 10295, 10556, 12285, 12561, 14308, 14569, 15287, 15355, 15431, 15497 and 15968) were analysed by sequencing using PCR primer pairs and published protocols [13]. The coding region mtDNA sequence was obtained for nine samples by amplifying five PCR fragments using the primer pairs L923 – H3108 (fragment A), L3073 – H5306 (fragment B), L5278 – H8861 (fragment C), L8799 – H12603 (fragment D) and L12572 – H15720 (fragment E) [13]. The PCR was carried out in 50-μl volumes, containing 1X Tris–HCl buffer, 200 μM of each dNTP, 2.5 mM MgCl_2_, 50 pmoles of each primer and 5 U of Taq polymerase (Bioline). The amplification was carried out in an Applied Biosystems 2720 Thermal Cycler with the following conditions: 30 amplification cycles with denaturation at 94°C for 30 s, annealing at different temperatures (56°C, 52°C, 55°C, 59°C and 57°C for A, B, C, D and E fragment respectively) for 30 s, and extension at 72°C for 4 min.

Sequencing reactions were performed using the BigDye Terminator Cycle Sequencing Kit v3.1 (Applied Biosystems). The HVR fragment was sequenced using L15676, H16401, L16340, H00408 and H00945 primers. The PCR fragments for SNP typing were sequenced with both forward and reverse primers. Finally, the coding region sequencing was carried out using both the amplification and internal primers. All the primers used for sequencing were previously described by Maca-Meyer et al.[13]. The sequencing products were run on an ABI 3130xl Genetic Analyser (Applied Biosystems) according to the manufacturer's recommendations. Sequences were analysed with the Sequencing Analysis software v 5.2 (Applied Biosystems) and manually inspected using FinchTV ver. 1.4.0 software (Geospiza, Inc.; Seattle, WA, USA; http://www.geospiza.com).

### Data analysis

The Mauritian sample was compared with its putative parental populations (South Asia, Madagascar, East Asia, Southeast Asia, Africa and Europe) and with other islands at the western fringe of the Indian Ocean (Réunion Island and Madagascar). The Reunionese sample from Berniell-Lee et al. (2008) [10] was used only for match analysis as it is not comparable with our miscellaneous sample from Mauritius due to founding events affecting its mtDNA diversity and its low representativeness of the whole population of Réunion [11]. The sample from the Dubut et al. (2009) study [11] could only be used for determining differences between Indian influences as it is based on the characterization of two concrete ethnic groups of Indian origin, the *Malbar* and the *Zarab*.

For comparison purposes, the mtDNA range used was 16065–16365 and all were reclassified into haplogroups according to their geographical adscription as in Dubut et al. 2009 [11]. Pair-wise F_ST_ genetic distances [15] based on haplogroup frequencies and diversity indices [16] were calculated as implemented in ARLEQUIN ver. 3.5.1.2 [17]. Multidimensional scaling (MDS) analysis of pair-wise F_ST_ distances was performed using the SPSS statistical program v.19 (SPSS, Inc.). Matches for Mauritian sequences with other areas were distributed following the most probable contributor hierarchical order, in such a way that, when a match occurred with India or Madagascar, it was removed from consideration as a match in other areas. The first hierarchical priority was given to India, following by Madagascar, sub-Saharan Africa and finally the remaining areas, according to demographic and historical data that place India as the main contributor followed by Malagasy and sub-Saharan African slaves [2]. The origin of the Mauritian putative Indian lineages was assigned using the Bayesian approach proposed by Mendizábal et al. 2008 [18]. The same method was applied to the analysis of African and Malagasy lineages with the aim of assessing the influence of the slave trade. For admixture analyses, m_y_ and m_L_ estimators were calculated using ADMIX 2.0 [19] and WLSAdmix (kindly provided by Dr. Jeffrey Long) [20] programs, respectively. The phylogenetic trees were constructed using median-joining networks as implemented in Network version 4.6.1.0 [21], and subsequently refined manually to resolve reticulations.

## Results

### Mauritian mtDNA complete sequences

All the Mauritian coding region sequences (GenBank accession numbers: KJ411336–KJ411423) could be assigned to 72 haplogroups and subhaplogroups (Table S3). However, to accomplish this affiliation, the complete mtDNA genome sequencing of nine samples (Figure S1) was necessary (GenBank accession numbers: KC577353–KC577361). Sample Ma-82 belongs to M5b haplogroup (with the 8784T defining mutation), samples Ma-70 and Ma-79 belong to the H13a2a haplogroup (sharing the 709, 1008, 2259 and 14872 diagnostic transitions), sample Ma-84 to the D4a subhaplogroup (sharing 152 3206 8473 14979 16129 diagnostic motif) and sample Ma-44, although it does not have the 16189 mutation, is classified as B4'5 as it has 8281–8289del. Some of these sequences have allowed us to define new subhaplogroups. Sample Ma-46 is classified as M42 (9156). This sequence defines a new branch of M42 [22], named M42b2 (Figure S2), and characterized by 1053 4197 15241 15562 and 16278 mutations. Sample Ma-66 is classified as R6 (Figure S3). This sequence redefines R6a1b subhaplogroup (12133 15067 16266!) and its sub-branches R6a1b1 (5894 16213) and R6a1b1a (15100), and also redefines the branch R6a1 (228 11075 14058 16274!), R6a1a (6305 8584 8650 16318 16320) and its sub-branch R6a1a1 (195! 373 961 7316). We also defined a new branch R6a2 (7202 9449 14470 16172). Ma-19 belongs to M49 haplogroup as it has 3780 and 16234 mutations; however, its further classification into subsequent clades remained unclear because it has the 10514 mutation in common with M49c, and the 11542 with M49d, but no other defining mutations for any subhaplogroup. Finally, sample Ma-12 is classified as M* as it does not share any subhaplogroup defining mutation.

### Phylogenetic analysis of Mauritian mtDNA lineages

In our dataset of 97 Mauritian sequences we detected 89 distinct haplotypes, 79 of which (81.44%) are unique to the sample. Measures of gene diversity indicate that Mauritian values (99.46% ± 0.24%) are within the range of its putative parental populations: South (99.61% ± 0.02%), East (99.43% ± 0.03%) and Southeast Asia (99.35% ± 0.03%), Madagascar (92.86% ± 1.03%) and Africa (99.42% ± 0.03%). The majority of Mauritian lineages are either Indian (M2, M3, M5, M6, R6, U7, U2, etc.) or African specific (L1c, L2a, L3b, L3d and L3e). There are also moderate frequencies of East or Southeast Asian lineages (B4, B5, D4a, F1a, etc.). Finally, the Mauritian sample contains some clear Malagasy (B4a1a, F3b, M7c3 and M23) as well as possible European types (K1, U3 and T2). The H13a2 samples have been assigned to an Indian influence, as H13 is a Near Eastern, not a European variant of haplogroup H [23]. Indeed, the H13a2a haplogroup have been observed in 5.9% of the Indian B'nei Israel community [24]. In the same vein, although in principle we have considered the K1, U3 and T2 lineages as a potential European input, the fact that two of those lineages are found both in India and Europe (the other lineage does not showed any match in the whole database) does call for caution. Furthermore, the only H lineages found in Mauritius belonging to the Near Eastern H13 haplogroup, when it is related to other H13 complete sequences, clusters with Indian, not European or Near Eastern, sequences (Figure S4). It is worth mentioning that H lineages account for 50% in continental Europe but the only ones detected in the Mauritian sample belong to an Indian branch, indicating a potential South Asian origin for these and other putative European lineages (K1, U3 and T2).

Furthermore, considering the importance of Madagascar as the labour source for Réunion and Mauritius, we should not rule out that some East African lineages present in Mauritian could be an indication of indirect gene flow from these regions through Madagascar. In this way, the 45% of the sub-Saharan lineages could be the result of direct gene flow from Madagascar. Additionally, the two Polynesian B4a1a1a samples from Mauritius carry the “Malagasy motif” (1473 3432A) described by Razafindrazaka et al. 2010 [6], confirming Madagascar as their origin. In summary, the Indian component in Mauritius could reach 58.76%, rendering the European contribution almost negligible (3.09%). The Malagasy, sub-Saharan African and East-Southeast Asian influences are then 16.60%, 10.21% and 11.34%, respectively.

**Figure 2:**
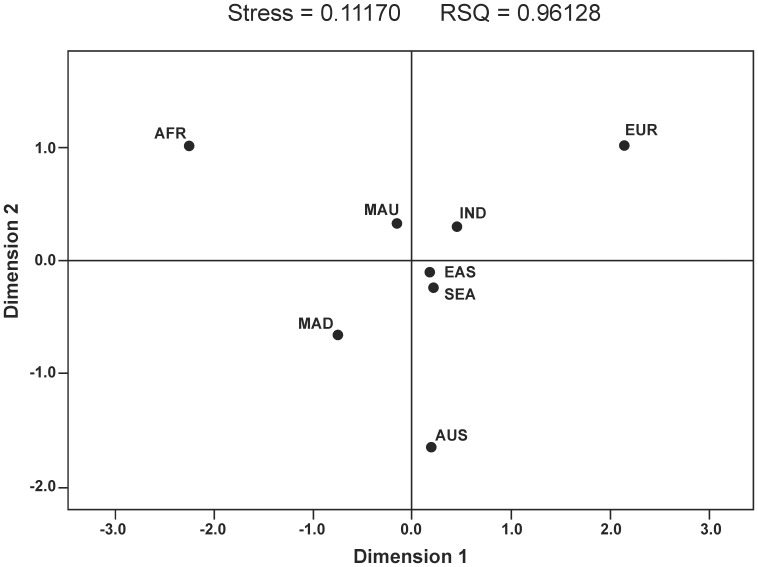
MDS plot based on haplogroup F_ST_ distances (MAU  =  Mauritius; MAD  =  Madagascar; IND  =  India; EAS  =  East Asia; SEA  =  Southeast Asia; AFR  =  Africa; AUS  =  Austronesia; EUR  =  Europe).

The Slatkin's F_ST_ distances (Table 1) between Mauritius and its putative parental populations are in agreement with the phylogenetic analysis (Figure 2). South Asia is the nearest Mauritian parental population (0.0183), followed by East and Southeast Asia (0.0330 and 0.0313, respectively), Madagascar (0.0414), and Africa (0.1185), with Europe (0.1366) being the farthest. Within South Asia (Table 2), the lower F_ST_ distance values have been shown to be with North India (0.0022), West India (0.0031) and Southeast India (0.0033).

**Table 1:** Linearized F_ST_ distances between Mauritius and its parental populations (MAU  =  Mauritius; MAD  =  Madagascar; IND  =  India; EAS  =  East Asia; SEA  =  Southeast Asia; AFR  =  Africa; EUR  =  Europe).

	MAU	MAD	IND	EAS	SEA	AFR	AUS	EUR
MAU	-	0.0414	0.0183	0.0330	0.0313	0.1185	0.1007	0.1366
MAD	0.0414	-	0.0720	0.0682	0.0617	0.1421	0.0554	0.2374
IND	0.0183	0.0720	-	0.0412	0.0414	0.1901	0.1126	0.1061
EAS	0.0330	0.0682	0.0412	-	0.0125	0.1818	0.0940	0.1526
SEA	0.0313	0.0617	0.0414	0.0125	-	0.1883	0.0871	0.1537
AFR	0.1185	0.1421	0.1901	0.1818	0.1883	-	0.2674	0.3352
AUS	0.1007	0.0554	0.1126	0.0940	0.0871	0.2674	-	0.2378
EUR	0.1366	0.2374	0.1061	0.1526	0.1537	0.3352	0.2378	-

**Table 2:** Linearized F_ST_ distances between Mauritius and the different South Asian sub-regions (MAU  =  Mauritius; PWI  =  Pakistan and West India; SWI  =  Southwest India; NI  =  North India; SEI  =  Southeast India; BEI  =  Bangladesh and East India).

	MAU	PWI	SWI	NI	SEI	BEI
MAU	-	0.0031	0.0082	0.0022	0.0033	0.0057
PWI	0.0031	-	0.0060	0.0015	0.0024	0.0051
SWI	0.0082	0.0060	-	0.0056	0.0052	0.0096
NI	0.0022	0.0015	0.0056	-	0.0018	0.0042
SEI	0.0033	0.0024	0.0052	0.0018	-	0.0058
BEI	0.0057	0.0051	0.0096	0.0042	0.0058	-

### Admixture analyses of the Mauritian population

Addressing exact haplotypic matches and linking these with the putative parental populations following the hierarchical order based on historical data (Table 3; Table S4), the highest value was found with South Asia (63.46%). Outside South Asia, the greatest number of matches was with Madagascar (15.38%), followed by East Asia (9.62%), Africa (7.69%), and Southeast Asia (3.85%). The European putative lineages have exact matches in India confirming that maternal contributions directly from Europe could be considered negligible. In fact, at present, there are no direct, unique, matches with the Europe database from the Mauritian sample.

**Table 3:** Summarized matches analysis results comparing Mauritius with its parental populations (IND  =  India; MAD  =  Madagascar; AFR  =  Africa; EAS  =  East Asia; SEA  =  Southeast Asia; EUR  =  Europe).

	Sample size	Total matches	Unique matches	Matches assignation following hierarchical order	Percentage
**IND**	6065	33	15	33	63.46%
**MAD**	170	9	1	8	15.38%
**AFR**	9780	17	4	4	7.69%
**EAS**	9074	19	2	5	9.62%
**SEA**	4293	15	2	2	3.85%
**EUR**	2913	13	0	0	0.00%
**Total**	**32295**	**52**	**24**	**52**	**100%**

More formal admixture measures based on haplogroup frequencies (Table 4) and using two distinct estimation programs (ADMIX 2.0, and WLSAdmix), show similar results to the phylogeographic analysis. The most important parental population for Mauritius is South Asia, with a contribution of 52.97%–48.47%. Other important contributions to the maternal lineages, which ostensibly derive from the slave trade, with admixture values of 23.07%–17.51% and 11.11%–7.20% are from Africa and Madagascar, respectively. The contribution of East and Southeast Asia is 10.12%–5.51% and 11.91%–5.91% respectively. Finally, the European input (3.82%–2.38%) is practically negligible. These contribution values based on haplogroup frequencies partly resemble the values obtained using haplotypic matches (Table 3). This indicates that our hierarchical order of most probable contributor, based on the historical data, provides congruent results, although it overestimated South Asian, and underestimated African, contributions respectively.

**Table 4:** Admixture estimations for Mauritian population (MAD  =  Madagascar; IND  =  India; EAS  =  East Asia; SEA  =  Southeast Asia; AFR  =  Africa; EUR  =  Europe).

	MAD	IND	EAS	SEA	AFR	EUR
m_L_ [20]	11.11±0.36%	52.97±0.54%	10.12±0.45%	5.91±0.40%	17.51±0.35%	2.38±0.27%
m_y_ [19]	7.20±0.63%	48.47±1.07%	5.51±1.08%	11.92±0.97%	23.07±0.61%	3.82±0.76%

### Interregional origin of Indian and African lineages in Mauritius

Previous research from Réunion Island [11], demonstrated that Southeast India was the main contributor to *Malbar* and *Zarab* ethnic group. In order to compare these results with that observed in Mauritius, we repeated the admixture calculation based on haplogroup frequencies with lineages of Indian adscription only. The major Indian contributor area to Mauritius was North India (49.23%). Other contributions came from Southeast (31.50%), and West India (19.26%). The contributions from Southwest and East India were negligible. The intraregional analysis of Mauritian haplotypic matches within India using a Bayesian estimator showed that Southeast (37.88% ± 0.99%) and North India (32.26% ± 1.02%) were the main manual labour origin source followed by West India (18.55% ± 1.21%), with Southwest (7.88% ± 1.05%) and East India (3.51% ± 1.04%) being minor contributors.

We also performed an intraregional analysis of sequences of African origin, which, indicated that African influence derived from Madagascar and East Africa (47.97% ± 2.39% and 26.40% ± 2.50%), with a West African input also detected (25.64% ± 2.44%). The admixture Bayesian estimator based on haplotypic matches corroborates Madagascar (76.75%) and East Africa (14.82%) as the main points of origin, with minor influences from West Central (5.25%) and West Africa (3.19%).

## Discussion

The analysis of complete genome sequences from Madagascar revealed an autochthonous linage named M23 [4], [25]. Our complete mtDNA study also discovered an unidentified M type that only shares the common M mutations. The presence of this rare lineage on Mauritius has to be the result of historical migrations, most probably from India or East-Southeast Asia. Future analysis within these regions should provide its most probably origin.

The mitogenomes presented here redefine R6a and M42b subhaplogroups. Haplogroup R6 is a clear Indian haplogroup associated with the demographic expansion event during an interglacial period before the Last Glacial Maximum [26]. We defined a new branch R6a1b1 based on our R6 Mauritian lineage within the R6a haplogroup previously defined by Chaubey et al. [27]. In order to determine if it is possible to track the precise origin of this lineage we determine its frequency in the different regions of India based on its HVRI motif (16129 16213 16266! 16274! 16362). The entire frequency of R6a1b1 in India is only 0.33% (Table S2). Within the different geographical areas the frequency is ∼0.30%, with the highest value in west India and Pakistan (0.48%) and the lowest in southwest India (0.15%). We also performed a network of the R6a1b1 HVRI data and observed that the Mauritian sample does not cluster with any Indian sample and splits directly from the basal motif (Figure S5). More phylogenetic analysis of R6 haplogroup would be necessary in the future to refine the R6 phylogenetic tree and determine the precise origin of the Mauritian sequence.

Although M42 was previously considered as an Australian aborigine lineage, complete sequences from relic tribes of India showed that the M42 haplogroup presents two branches with clear geographical adscription [22]. M42a is restricted to aboriginal people from Australia, whereas M42b is a clear Indian lineage. The coalescence time estimated for the divergence of M42 Indian and Australian lineages (∼55 Kya) is consistent with the archaeological evidence regarding the first human arrival to Australia. Our Mauritian sequence defines a new branch M42b2 within the Indian M42 branch (Figure S2) reinforcing the importance of Indian ancestry in Mauritius. As the M42b2 HVRI motif is defined only by mutations common to other haplogroups (16189 16223 16278) it was discarded to obtain conclusions from HVRI data (16065–16385).

Historically, the parental populations of Mauritius have been identified as deriving from India, sub-Saharan Africa, Madagascar, China and Europe [2]. The complex demographic history of Mauritius is clearly reflected by its high genetic diversity (99.46%) that could be explained by the successive founder events motivated by labour sourcing initiated by the different European colonizers: Dutch, French and British. The complexity of Mauritius' demography is also reflected in its diverse genetic composition. The important migration of indentured workers brought from India is confirmed by the high frequency of Indian-specific lineages in the Mauritian maternal genetic pool. Furthermore, all the data analysis comparing Mauritius with its putative parental populations corroborates that India is the main source of Mauritian lineages. In fact, the majority of lineages from Mauritius were classified as Indian-specific haplogroups, reaching 58.76%, with the island showing its lowest value for Slatkin's F_ST_ genetic distance (Table 1) with this region. Furthermore, the matches and admixture analyses place India as the main contributor with 64.3% of exact matches and an averaged contribution of approximately 50%.

Focusing on the Indian lineages, the lower F_ST_ values of the Mauritian sample derived from North, West and Southeast India (Table 2). As expected, these regions also show the highest percentage of exact matches and the higher genetic contribution within India. In fact, admixture analysis suggests that the Indian specific lineages could be explained solely by North, Southeast and West India contributions (49.23%, 31.50% and 19.26%, respectively). This result is congruent with the historical record of worker recruitment in India [28]. It is known that Calcutta (North India) was an important port of embarkation of indentured labourers to Mauritius between 1834 and 1910. By the middle of the 19^th^ century, Bihar (North India) had become the centre of recruitment. Many labourers also came from other specific regions of the Northwestern provinces. The apparently better physical constitution of South Indian workers, and lower mortality rate during transportation, made this region another important source of labourers [28].

The other significant contributor to the Mauritian population derived from the historic slave trade, both from Africa and Madagascar. The values of exact matches (15.4%) and the admixture values (11–7%) indicate that Madagascar provided a remarkable amount of maternal lineages to Mauritius. In the same manner, 7.7% of exact matches and a contribution estimation of approximately 20% evidence the existence of a direct sub-Saharan slave trade from the continent. These results emphasize the contribution of Malagasy and sub-Saharan slaves to the modern Mauritian gene pool in spite of high mortality within these populations from hard working conditions and disease. A more refined admixture analyses based on haplogroup frequencies and haplotypic matches revealed a more significant contribution from Madagascar and East Africa (75%–90%) whereas a lower percentage come from West Africa (10%–25%). The differences in Malagasy-sub-Saharan Africa contributions using exact matches and Bayesian estimations, and those obtained using admixture calculations based on haplogroup frequencies seems to be caused by the presence of Malagasy-specific haplotypes on Mauritius and their absence on the African continent, slightly overestimating the real contribution of Madagascar.

The genetic impact of manual workers, and in later years merchants, from China is also detected by the presence of East Asian lineages. East and Southeast Asia showed a percentage of 9.6% and 3.9% of exact matches with the Mauritian sample, respectively. The admixture analysis points to an estimated genetic contribution of 10.0–5.5% and 11.9–5.9% for East and Southeast Asia respectively.

Finally, although French and British colonizers settled Mauritius, only a 2.4–3.8% European contribution has been detected. This could be explained by the fact that European colonizers were mainly men and the few European females did not mix with local males.

Mauritius and Réunion had similar historical backgrounds and the same putative parental populations and this is reflected in the match analysis. The two multi-ethnic populations present exact haplotypic matches in lineages present both in India (10) and Madagascar/Africa (5) (Table S4). Mauritius and Réunion share one haplotypic match in one lineage of the M43b Indian haplogroup [29], [30] that has not been observed in the parental population. This result highlights the similarities of their genetic histories. However, evaluations of the exact origin of Indian lineages also reveal important differences. Dubut et al. [11] determine the origin of the Indian lineages of the *Malbar* and *Zarab* ethnic groups, as well as other haplotypes with South Asian origin and conclude that these lineages were contributed mainly from Southeastern India (63%). For Mauritius, an important portion of lineages came from Northern India, whereas only 15% of Reunionese Indian descendants derive from the North. This points to differences in the main recruitment centres of indentured labourers for Mauritius and Réunion, probably due to the fact that they belonged to Britain and France, respectively. After the abolition of slavery in 1848, the French administration for Réunion started to recruit workers from India, primarily from the colony in Pondicherry, located in Southeast India, and this was likely the principal area of origin of indentured labourers brought to Réunion Island [31], [32], [33]. France reached a convention with the British that allowed the recruitment of 6,000 Indian indentured labourers per year. Between 1848 and 1860, around 40,000 Indian manual labourers were brought to Mauritius, most of them originating from Tamil Nadu. Curiously, the results from Dubut et al. indicates that Andhra Pradesh contributed more to Réunion than Tamil Nadu, contradicting the historical records.

From a genetic perspective, it is clear that the diverse human movements around the Indian Ocean have shaped the demographic composition of the islands situated at its western edge. Madagascar and the Comoros archipelago's genetic compositions have mainly been determined by ancient human migrations from Austronesia and East Africa, a result of the Bantu expansion. The Mascarene archipelago, although uninhabited at the time of its discovery, show a complex demographic structure due to continuous waves of human migration motivated by the need for manual labour during European expansion. Although the French and British controlled these islands, their genetic impact was insignificant, at least from the mtDNA point of view.

Given both the location and role as a ‘labour island’, a key focus of future research should be archaeological samples, as performed on other admixed populations [34], [35], [36]. This should aim to investigate differential geneflow over time, their admixture, and sex-specific dichotomy. The comparison of ancient DNA results with those obtained from current populations would provide valuable information regarding the temporal evolution of human populations in the island.

Finally, the present research potentially has implications beyond assessment of the island's past. Modern populations on Mauritius have a remarkably high prevalence of specific non-communicable diseases, particularly type 2 diabetes. Determining the genetic structure of small island populations, such as Mauritius, is the first step to forging links between the molecular causes of illnesses and their impacts on specific populations, particularly as these diseases appear to have significantly greater relative impact on these small communities.

## Supporting Information

Figure S1 **Phylogenetic tree of complete Mauritian sequences.** Number along links refers to nucleotide changes, whereas “d” and “i” indicate deletions and insertions, respectively. GenBank accessions and geographic origin are referred for each complete sequence. Red numbers correspond to recurrent mutations (309iC, 315iC, 522dCA, 523iCA, 16182C, 16183C and 16519) not taken into account during tree construction. Exclamation marks indicate retromutations. Mutations highlighted in light blue correspond to newly defined or redefined branches.
(TIF)

Figure S2 **Phylogenetic tree of complete haplogroup M42 sequences.** Codes as in Figure S1.
(TIF)

Figure S3 **Phylogenetic tree of complete haplogroup R6a sequences.** Codes as in Figure S1. Underlined numbers correspond to recurrent mutations within R6 haplogroup.
(TIF)

Figure S4 **Phylogenetic tree of complete haplogroup H13a2 sequences.** Codes as in Figure S1.
(TIF)

Figure S5 **HVRI network of R6 sequences.** Star corresponds to CRS haplotype. Codes as in Figure S1.
(TIF)

Table S1 **Populations used for comparison in this study.**
(XLSX)

Table S2 **Haplogroup frequencies for the comparison database (MAU  =  Mauritius; MAD  =  Madagascar; IND  =  India; PWI  =  Pakistan and West India; SWI  =  Southwest India; NI  =  North India; SEI  =  Southeast India; BEI  =  Bangladesh and East India; EAS  =  East Asia; SEA  =  Southeast Asia; AFR  =  Africa; WAF  =  West Africa; EAF  =  East Africa; AUS  =  Austronesia; EUR  =  Europe).**
(XLSX)

Table S3 **Partial (HVRI and HVRII) mtDNA haplotypes, haplogroups and coding region mutations observed for the Mauritian samples analyzed in this study.** The GenBank accesion numbers are indicated in the last column.
(XLSX)

Table S4 **Detailed matches information (MAU  =  Mauritius; REU  =  Réunion Island; MAD  =  Madagascar; IND  =  India; PWI  =  Pakistan and West India; SWI  =  Southwest India; NI  =  North India; SEI  =  Southeast India; BEI  =  Bangladesh and East India; EAS  =  East Asia; SEA  =  Southeast Asia; AFR  =  Africa; WAF  =  West Africa; EAF  =  East Africa; EUR  =  Europe).**
(XLSX)
