# Supplementary figures and images for: Multiple Ethnic Origins of Mitochondrial DNA Lineages for the Population of Mauritius
Source: PLoS One. 2014 Mar 27;9(3):e93294. doi: 10.1371/journal.pone.0093294 (PMC3968120; doi:10.1371/journal.pone.0093294)

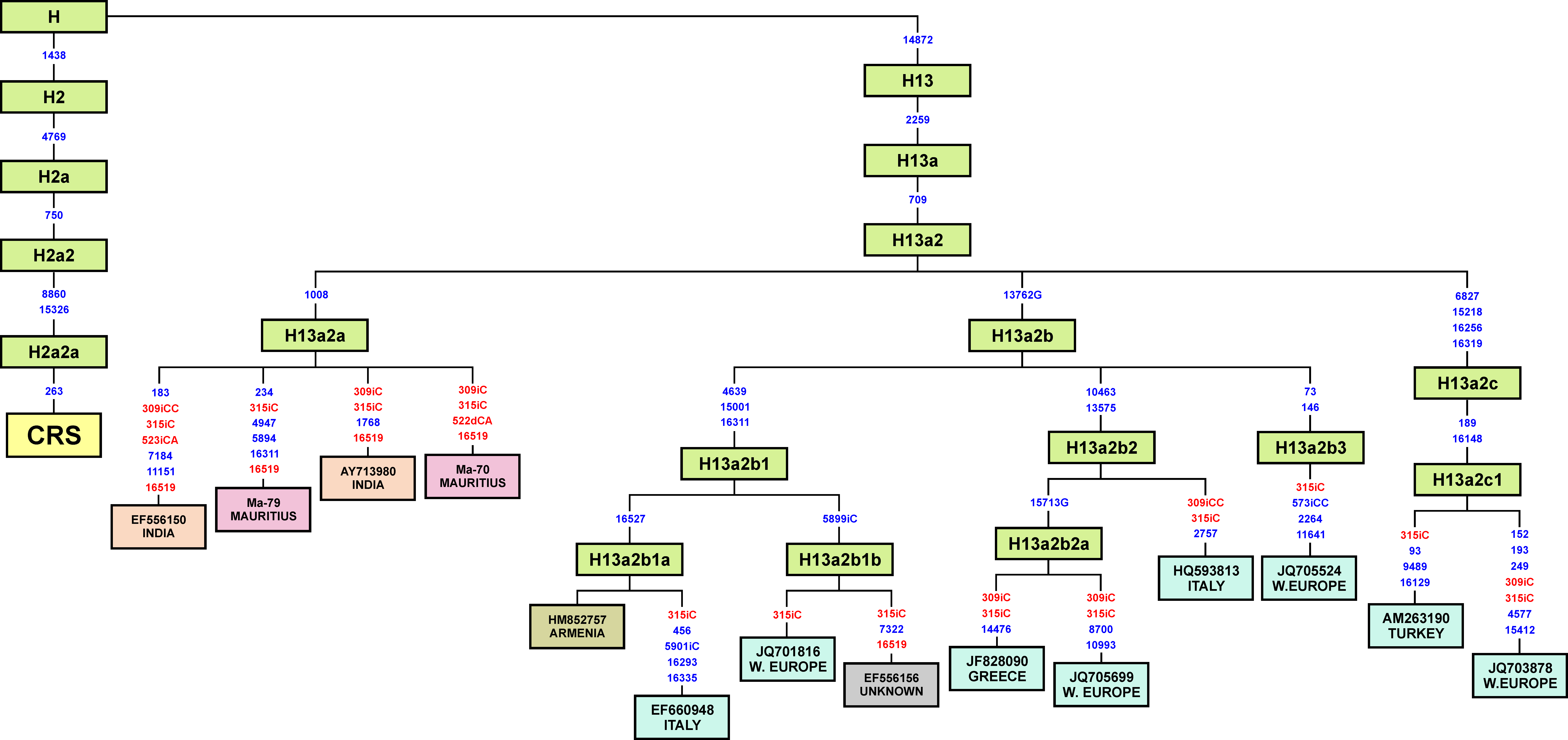

Supplement: Figure S4 — Phylogenetic tree of complete haplogroup H13a2 sequences. Codes as in Figure S1. (TIF) [file pone.0093294.s004.tif]

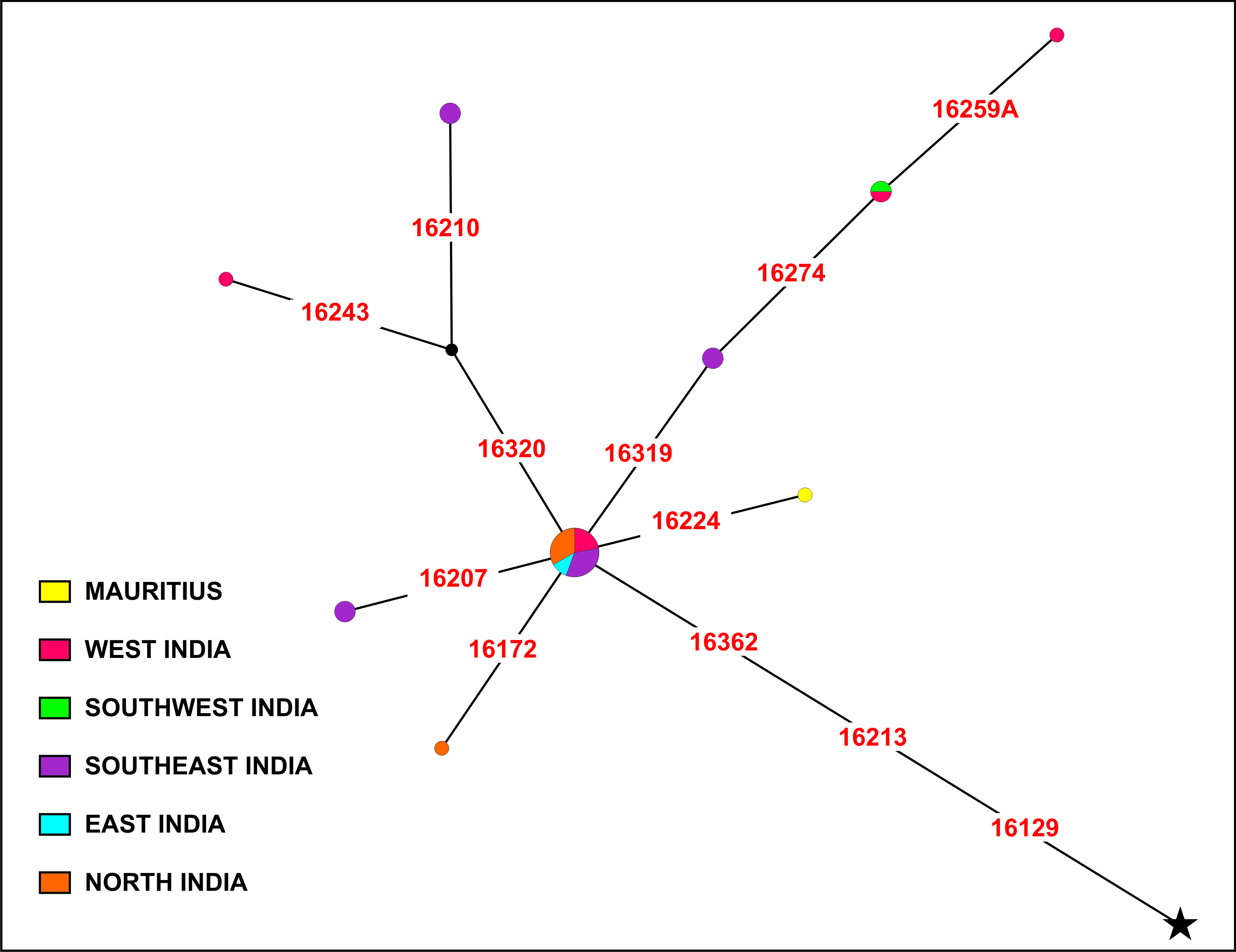

Supplement: Figure S5 — HVRI network of R6 sequences. Star corresponds to CRS haplotype. Codes as in Figure S1. (TIF) [file pone.0093294.s005.tif]
